# A real-world analysis of the influence of age on maintenance hemodialysis patients: managing serum phosphorus with sucroferric oxyhydroxide as part of routine clinical care

**DOI:** 10.1007/s11255-022-03327-w

**Published:** 2022-08-11

**Authors:** Connie M. Rhee, Meijiao Zhou, Rachael Woznick, Claudy Mullon, Michael S. Anger, Linda H. Ficociello

**Affiliations:** 1 Harold Simmons Center for Kidney Disease Research and Epidemiology, Division of Nephrology, Hypertension and Kidney Transplantation, University of California Irvine, Orange, CA USA; 2 Global Medical Office, Fresenius Medical Care, 920 Winter Street, Waltham, MA 02451 USA; 3 Fresenius Kidney Care, Centre Point, West Allis, WI USA

**Keywords:** Sucroferric oxyhydroxide, Hemodialysis, Age, Phosphate binder, Pill burden, Phosphorus

## Abstract

**Objective:**

Despite the growing number of elderly hemodialysis patients, the influence of age on nutritional parameters, serum phosphorus (sP), and use of phosphate-binder (PB) medications has not been well characterized. We aimed to describe age-related differences in patient characteristics in a large, real-world cohort of maintenance hemodialysis patients, and to examine the impact of age on sP management with sucroferric oxyhydroxide (SO).

**Methods:**

We retrospectively analyzed de-identified data from 2017 adult, in-center hemodialysis patients who switched from another PB to SO monotherapy as part of routine clinical care. Changes in baseline PB pill burden, sP levels, and nutritional and dialytic clearance parameters were assessed across varying age groups through 6 months.

**Results:**

At baseline, older patients had lower mean sP, serum albumin, and pre-dialysis weights compared with younger patients. Prescription of SO was associated with a 62% increase in the proportion of patients achieving sP ≤ 5.5 mg/dl and a 42% reduction in daily pill burden. The proportion of patients achieving sP ≤ 5.5 mg/dl after transitioning to SO increased by 113, 96, 68, 77, 61, 37 and 40% among those aged 19–29, 30–39, 40–49, 50–59, 60–69, 70–79, and ≥ 80 years, respectively.

**Conclusions:**

Older patients had worse nutritional parameters, lower pill burden, and lower sP at baseline versus younger counterparts. Prescription of SO was associated with improved sP control and reduced pill burden across all ages.

**Supplementary Information:**

The online version contains supplementary material available at 10.1007/s11255-022-03327-w.

## Introduction

The population of patients requiring dialysis continues to grow rapidly, largely attributed to the combination of the aging global population and increasing rates of key risk factors such as diabetes and hypertension [1]. The elderly represent the fastest growing segment of the dialysis population, accounting for 25% to 30% of dialysis patients in end-stage kidney disease (ESKD) registries [1–3]. This aging phenomenon amplifies the need to understand age-related differences in the dialysis population. To that end, age-related differences in comorbidities, nutritional status, and associated mortality risk have been observed in this population [3–7]. Compared with their younger counterparts, elderly dialysis patients have been reported to have worse nutritional parameters suggestive of protein energy malnutrition [5–7], impaired physical function [8, 9], lower health-related quality of life (HRQoL) [10], and high morbidity and mortality rates [11].

Hyperphosphatemia is of particular importance in dialysis patients across all ages, given its contribution to both bone and cardiovascular complications in this population [12, 13]. In addition to experimental evidence linking hyperphosphatemia to coronary artery calcification [14], hyperphosphatemia has been independently associated with cardiovascular disease and mortality in patients with chronic kidney disease (CKD), particularly those on dialysis [15–17]. Limited data suggest that age may influence the association between serum phosphorus and outcomes in hemodialysis patients, possibly owing to the lower serum phosphorus levels seen in elderly patients [3]. However, the influence of age on serum phosphorus levels (sP) in this population has not been well characterized.

Current Kidney Disease Improving Global Outcomes (KDIGO) guidelines recommend lowering serum phosphorus levels toward the normal range in patients with CKD on dialysis through dietary restriction, intensifying dialysis schedules, and/or phosphate-binding (PB) medications [18]. Given that dialysis alone may not be sufficient in achieving target sP levels [19], most hemodialysis patients with CKD require PB medications to enhance phosphate elimination [12, 20]. Indeed, current data from the Dialysis Outcomes and Practice Patterns Study (DOPPS) indicate that PB medications are prescribed in over 80% of hemodialysis patients [21]. Despite this, over 40% of these patients have sP levels over 5.5 mg/dl [22]. This finding is often attributed to nonadherence, a problem reported in up to 62% of hemodialysis patients that is associated, at least in part, with the high pill burden of these therapies [23, 24]. PBs account for half of the pill burden in maintenance dialysis patients, which often exceeds 20 pills per day [24]. Furthermore, pill burden from PBs has been associated with lower adherence rates, higher serum phosphorus and parathyroid hormone (PTH) levels, and lower HRQoL scores in hemodialysis patients. [24–26]

Sucroferric oxyhydroxide (SO; VELPHORO^®^ [Fresenius Medical Care Renal Therapies Group, Waltham, MA, USA]) is an iron-based, noncalcium chewable PB indicated for treatment of hyperphosphatemia in patients with CKD on dialysis. In randomized, controlled studies, this agent lowered sP comparable to sevelamer but with a lower pill burden [27, 28]. Similarly, effective phosphate-lowering coupled with a lower pill burden has been shown in a number of real-world settings when SO is prescribed as a part of routine therapy [29–32].

Despite unique considerations in the clinical management of elderly patients with CKD [2, 11], few studies have focused on age-related differences in patient, treatment, and biochemical characteristics of the dialysis population. We aimed to describe age-related differences in patient characteristics in a large, national real-world cohort of patients on maintenance hemodialysis and to examine the impact of age on the effects on sP management with SO.

## Methods

This retrospective study used de-identified electronic medical records extracted from Fresenius Kidney Care (FKC) clinical data warehouse, and prescription fill information retrieved from FreseniusRx, a renal pharmacy service, and medical review data. Adult patients (age ≥ 18 years) who received in-center hemodialysis from FKC who switched from another PB to SO monotherapy between May 2018 and May 2019 were included in the study. Patients were eligible for inclusion if they had been on PB monotherapy for at least 3 months before SO prescription and had sP measured in the month before SO start and in at least 5 out of the 6 months during SO monotherapy treatment. Observation periods were defined as quarterly intervals, including the baseline (BL, 3 months before SO prescription) and SO follow-up (Q1 and Q2; 1–3 and 4–6 months of SO treatment, respectively) periods.

### Study endpoints and assessments

Patient-level demographic characteristics (age, sex, race, ethnicity), pre/post-dialysis weight, body mass index (BMI), dialysis vintage, primary cause of kidney failure, and PB use were evaluated at baseline. Data on primary cause of kidney failure were collected by the dialysis clinic staff when they completed the ESRD history assessments using information provided by the physicians. Clinical variables of interest included the following parameters: serum mineral bone disorder (MBD) markers (phosphorus, calcium, and intact PTH [iPTH]); nutritional and dialytic clearance parameters (serum albumin, body weight, single pool normalized protein catabolic rate [spnPCR], and single pool Kt/V [spKt/V]); anemia and iron indices (ferritin, transferrin saturation [TSAT], and hemoglobin); and dose and use of MBD medications and anemia pharmacotherapies. Data on phosphorus-attuned albumin was also collected, in which serum albumin was divided by sP, to assess the impact of lowering sP without the unintended consequence of restricting dietary protein intake and lowering serum albumin levels [30].

Blood samples were drawn generally on the same day of each week, using standardized methods at FKC facilities, and analyzed at a central laboratory (Spectra Laboratories, Rockleigh, NJ, USA). Laboratory tests were measured monthly except for hemoglobin which was measured weekly, and serum ferritin and iPTH, which were measured quarterly per standard practice at FKC clinics. The use and medications doses (PB binders, cinacalcet, vitamin D, intravenous [IV] iron sucrose, and erythropoiesis-stimulating agent [ESA]) and the proportion of patients within the upper sP limit (sP ≤ 5.5 mg/dl per National Kidney Foundation Kidney Disease Outcomes Quality Initiative [NKF KDOQI] recommendations and sP ≤ 4.5 mg/dl per KDIGO guidelines) were evaluated quarterly. This study was reviewed by New England Independent Review Board (NEIRB) and determined to be exempted under the Common Rule and applicable guidance.

### Statistical analysis

Patients were categorized into seven age groups (19–29, 30–39, 40–49, 50–59, 60–69, 70–79, and ≥ 80 years old) to assess the impact of age on the management of sP. Baseline characteristics were presented as mean ± SD for continuous variables and number of patients (percentage) for categorical variables. Analysis of variance (ANOVA) and Chi-square were carried out to examine the differences of baseline variables across age groups. Mean laboratory measures and medication usage were averaged for each patient. Quarterly means of continuous data were calculated using mixed effects linear regression. Summary statistics were presented as least-squared means (standard errors [SE]) with comparison across treatment periods (overall *P* values) and comparisons between baseline and follow-up periods. Cochran’s *Q* test and McNemar chi-square test were employed for significance testing of categorical variables. Percentages of patients achieving sP ≤ 5.5 mg/dl and PB pills were depicted in figures. Two-tailed *P* values < 0.05 were considered as statistically significant. All analyses were performed stratified by age groups and were conducted with SAS (version 9.4; SAS Institute Inc, Cary, NC, USA).

## Results

### Patient characteristics overall and across age groups

Demographic and baseline characteristics of the study population are shown in Table 1. Mean ± SD patient age in the overall population was 56.4 ± 13.4 years. The proportion of patients across the age categories was 3.2% (19–29 years), 8.4% (30–39 years), 17.9% (40–49 years), 26.9% (50–59 years), 27.4% (60–69 years), 12.2% (70–79 years), and 3.9% (≥ 80 years). The overall population was comprised of 39.3% women, and 37.3 and 13.0% were African American and Hispanic, respectively. The proportions of African American and Hispanic patients were highest among younger patients (44.6 and 16.9%, respectively, in those aged 19–29 years) and declined with age (24.1 and 8.9%, respectively, in those ≥ 80 years). The mean dialysis vintage was 3.9 ± 3.8 years, with shorter vintage observed among patients in the 19–29 years age group (3.3 ± 2.8 years).

**Table 1:** Comparison of baseline characteristics among age groups

Characteristics	All patients (*n* = 2017)	19–29 years (*n* = 65)	30–39 years (*n* = 169)	40–49 years (*n* = 362)	50–59 years (*n* = 542)	60–69 years (*n* = 553)	70–79 years (*n* = 247)	80+ years (*n* = 79)	*P* value
Age (years)	56.4 ± 13.4	25.2 ± 2.8	35.2 ± 2.8	45.2 ± 2.9	54.7 ± 2.9	64.1 ± 2.7	73.5 ± 2.9	82.9 ± 2.5	
Dialysis vintage (years)	3.9 ± 3.8	3.3 ± 2.8	4.3 ± 4.3	4.0 ± 4.0	3.9 ± 4.1	3.9 ± 3.6	4.0 ± 3.7	3.1 ± 2.9	0.36
Pre-dialysis weight (kg)	91.3 ± 26.1	80.8 ± 29.1	93.8 ± 30.7	99.2 ± 29.6	93.5 ± 25.0	90.6 ± 23.5	82.4 ± 21.2	75.4 ± 17.5	< 0.0001
Post-dialysis weight (kg)	88.7 ± 25.6	78.4 ± 28.8	90.9 ± 30.3	96.3 ± 29.1	90.9 ± 24.5	88.2 ± 23.0	80.3 ± 20.8	73.5 ± 17.2	< 0.0001
BMI (kg/m^2^)	30.7 ± 8.1	27.5 ± 8.3	30.6 ± 8.9	32.8 ± 9.2	31.1 ± 7.6	30.7 ± 7.6	28.8 ± 7.1	26.5 ± 4.9	< 0.0001
Female, *n* (%)	793 (39.3)	30 (46.2)	63 (37.3)	125 (34.5)	183 (33.8)	230 (41.6)	120 (48.6)	42 (53.2)	< 0.0001
Race, *n* (%)									0.002
White	890 (44.1)	23 (35.4)	70 (41.4)	138 (38.1)	223 (41.1)	279 (50.5)	115 (46.6)	42 (53.2)	
African American	752 (37.3)	29 (44.6)	69 (40.8)	162 (44.8)	202 (37.3)	187 (33.8)	84 (34.0)	19 (24.1)	
Other	61 (3.0)	3 (4.6)	7 (4.1)	6 (1.7)	20 (3.7)	12 (2.2)	7 (2.8)	6 (7.6)	
Unknown	314 (15.6)	10 (15.4)	23 (13.6)	56 (15.5)	97 (17.9)	75 (13.6)	41 (16.6)	12 (15.2)	
Ethnicity, *n* (%)									0.23
Hispanic	263 (13.0)	11 (16.9)	23 (13.6)	45 (12.4)	74 (13.7)	82 (14.8)	21 (8.5)	7 (8.9)	
Not Hispanic	1397 (69.3)	45 (69.2)	124 (73.4)	251 (69.3)	359 (66.2)	384 (69.4)	177 (71.7)	57 (72.2)	
Unknown	357 (17.7)	9 (13.9)	22 (13.0)	66 (18.2)	109 (20.1)	87 (15.7)	49 (19.8)	15 (19.0)	
Primary cause of kidney failure, *n* (%)									< 0.0001
Diabetes mellitus	963 (47.7)	5 (7.7)	58 (34.3)	157 (43.4)	283 (52.2)	296 (53.5)	125 (50.6)	39 (49.4)	
Hypertension	675 (33.5)	22 (33.8)	58 (34.3)	126 (34.8)	180 (33.2)	168 (30.4)	90 (36.4)	31 (39.2)	
Glomerulonephritis	108 (5.4)	12 (18.5)	19 (11.2)	22 (6.1)	20 (3.7)	23 (4.2)	9 (3.6)	3 (3.8)	
Polycystic/hereditary/congenital diseases	49 (2.4)	4 (6.2)	5 (3.0)	14 (3.9)	8 (1.5)	13 (2.4)	4 (1.6)	1 (1.3)	
Transplant complications	60 (3.0)	3 (4.6)	9 (5.3)	16 (4.4)	14 (2.6)	15 (2.7)	3 (1.2)	0 (0.0)	
Secondary glomerulonephritis/vasculitis	42 (2.1)	8 (12.3)	12 (7.1)	7 (1.3)	7 (1.3)	3 (0.5)	4 (1.6)	1 (1.3)	
Other/unknown^a^	120 (6.0)	11 (16.9)	8 (4.7)	20 (5.5)	30 (5.5)	35 (6.3)	12 (4.9)	4 (5.1)	
Phosphate binder at baseline, *n* (%)									0.053
Sevelamer	826 (41.0)	17 (26.2)	63 (37.3)	160 (44.2)	215 (39.7)	210 (38.0)	129 (52.2)	32 (40.5)	
Calcium acetate	844 (41.8)	32 (49.2)	70 (41.4)	142 (39.2)	237 (43.7)	243 (43.9)	86 (34.8)	34 (43.0)	
Lanthanum carbonate	61 (3.0)	2 (3.1)	3 (1.8)	15 (4.1)	11 (2.0)	21 (3.8)	6 (2.4)	3 (3.8)	
Ferric citrate	133 (6.6)	6 (9.2)	13 (7.7)	23 (6.4)	39 (7.2)	36 (6.5)	12 (4.9)	4 (5.1)	
Switch among binders^b^	153 (7.6)	8 (12.3)	20 (11.8)	22 (6.1)	40 (7.4)	43 (7.8)	14 (5.7)	6 (7.6)	

Summary estimates are presented as mean ± standard deviation or number (percent) of patients; *P* value shows the differences among age categories

*BMI* body mass index

^a^Included 3 unknown causes in overall patients (*n* = 1 in group of age 50–59; *n* = 2 in group of age 60–69)

^b^Among 153 patients, the most frequently used phosphate binders were sevelamer (46.4%, *n* = 71), calcium acetate (31.4%, *n* = 48), ferric citrate (16.3%, *n* = 25) and lanthanum carbonate (5.9%, *n* = 9)

**Fig. 1:**
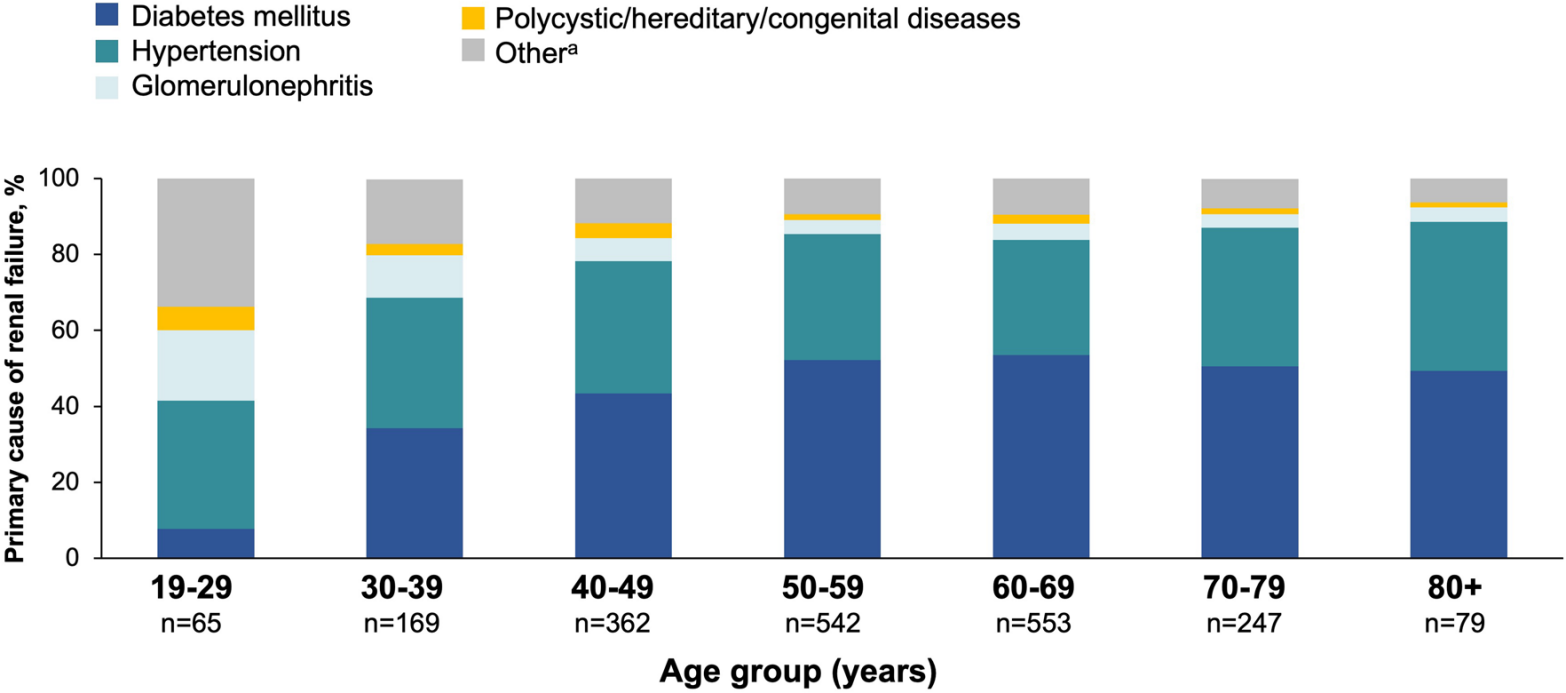
Etiology of kidney failure by age group. ^a^Includes transplant complications, secondary glomerulonephritis/vasculitis, and other/unknown causes

The primary cause of kidney failure differed across age groups, with diabetes accounting for fewer than 10% of cases in the 19–29 years age group, but approximately half of cases in patients ≥ 50 years old (Fig. 1). Hypertension accounted for a larger proportion of cases among younger patients, accounting for over one-third of cases in those aged 19–29 years. Additionally, congenital/hereditary/polycystic diseases and secondary glomerulonephritis were more common etiologies of CKD in younger patients. There were also differences in baseline PB use across age (Table 1). Whereas a similar proportion of patients were prescribed sevelamer vs. calcium acetate in the overall cohort (41.0 vs. 41.8%, respectively), there was a two-fold higher prevalence of prescription of calcium acetate vs. sevelamer use in the youngest (19–29 years) age group (49.2 vs. 26.2%, respectively). With respect to other clinical characteristics, pre- and post-dialysis weights and BMI were highest among patients aged 30–69 and were lowest among the youngest and oldest age groups.

### Serum phosphorus before and after SO across age

**Fig. 2:**
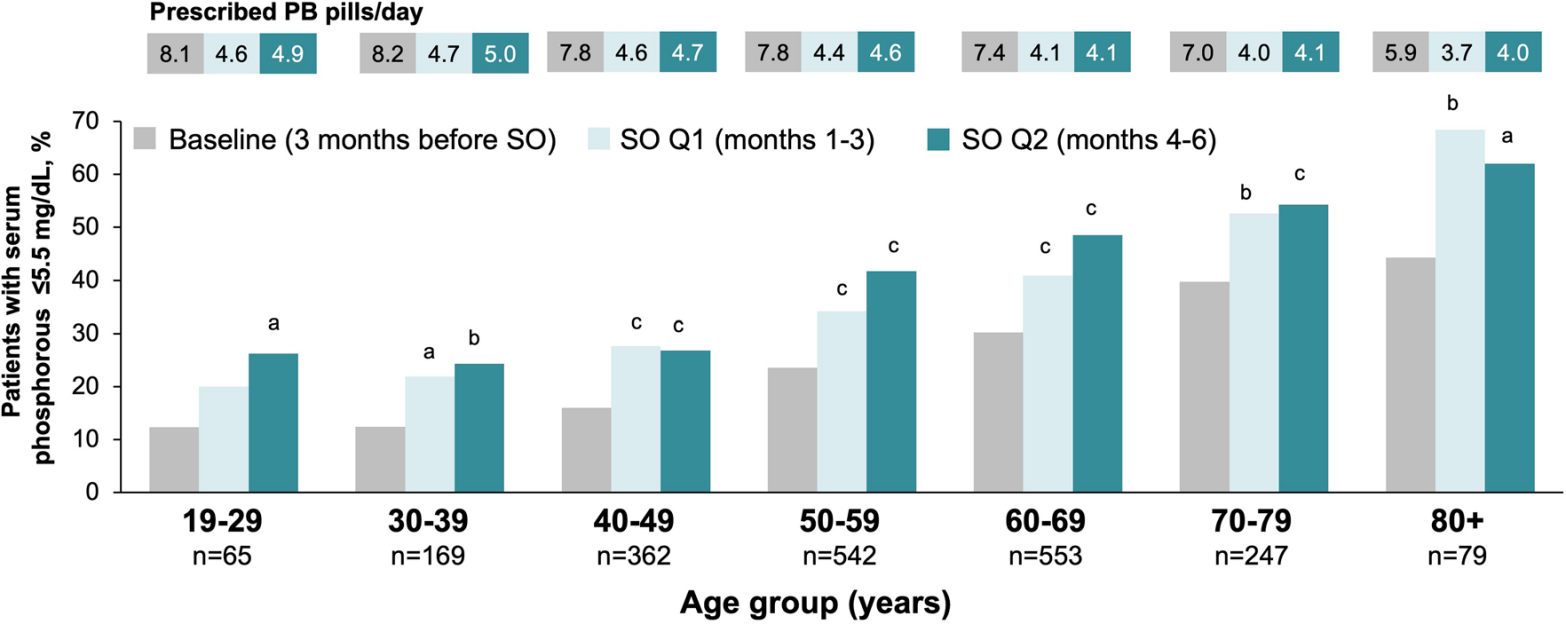
Change in percentage of patients with sP ≤ 5.5 mg/dl from baseline to Q1 (3 months) and Q2 (6 months) after switch to SO by age group. ^a^*P* < 0.05 vs. baseline; ^b^*P* < 0.01 vs. baseline; ^c^*P* < 0.001 vs. baseline. *PB* phosphate binder, *SO* sucroferric oxyhydroxide

At baseline, younger patients were less likely to have sP ≤ 5.5 mg/dl compared with patients 60 years and older. The highest proportion of patients with sP ≤ 5.5 mg/dl at baseline was observed in patients 80 years and older (44.3%), compared with only 12.3% of patients aged 19–29 years. After switching to SO, significant reductions in sP and increases in the proportion of patients with sP ≤ 5.5 mg/dl were observed across all age groups (Table 2, Fig. 2). The greatest relative increase in the proportion of patients with sP ≤ 5.5 mg/dl occurred in younger patients. The relative increases in % with sP ≤ 5.5 mg/dl from baseline to Q2 after switching to SO were 113, 96, 68, 77, 61, 37 and 40% for younger to older groups. Significant increases in the proportion of patients achieving sP ≤ 4.5 mg/dl were observed in the overall population after switching to SO (Table 2). Significantly more patients 40 years of age and older achieved sP ≤ 4.5 mg/dl 4 to 6 months (Q2) after switching to SO.

**Table 2:** Comparison of changes in MBD markers and PB pill burden among age groups

Measure	Period	All patients (*n* = 2017)	19–29 years (*n* = 65)	30–39 years (*n* = 169)	40–49 years (*n* = 362)	50–59 years (*n* = 542)	60–69 years (*n* = 553)	70–79 years (*n* = 247)	80+ years (*n* = 79)
P (mg/dl)	BL	6.39 (0.03)	7.11 (0.19)	6.99 (0.11)	6.77 (0.07)	6.50 (0.06)	6.13 (0.05)	5.82 (0.07)	5.73 (0.12)
	Q1	6.13 (0.03)^c^	6.96 (0.19)	6.79 (0.11)^a^	6.51 (0.07)^c^	6.17 (0.06)^c^	5.92 (0.05)^c^	5.61 (0.07)^b^	5.21 (0.12)^c^
	Q2	6.00 (0.03)^c^	6.76 (0.19)^a^	6.68 (0.11)^c^	6.49 (0.07)^c^	6.03 (0.06)^c^	5.70 (0.05)^c^	5.51 (0.07)^c^	5.20 (0.12)^c^
	*P*^d^	< 0.0001	0.03	0.0004	< 0.0001	< 0.0001	< 0.0001	< 0.0001	< 0.0001
sP ≤ 5.5 mg/dl (%)	BL	25.5	12.3	12.4	16	23.6	30.2	39.7	44.3
	Q1	36.9^c^	20	21.9^a^	27.6^c^	34.1^c^	40.9^c^	52.6	68.4^b^
	Q2	41.2^c^	26.2^a^	24.3^b^	26.8^c^	41.7^c^	48.5^c^	54.3^c^	62.0^a^
	*P*^d^	< 0.0001	0.047	0.001	< 0.0001	< 0.0001	< 0.0001	< 0.0001	0.0006
sP ≤ 4.5 mg/dl (%)	BL	8.2	3.1	3.6	4.1	9.6	9	13	11.4
	Q1	12.3^c^	3.1	5.3	6.4	12.4	14.6^a^	19^a^	25.3^a^
	Q2	15.4^c^	9.2	6.5	8.6^a^	14.8^a^	19.9^c^	20.2^a^	27.8^a^
	*P*^d^	< 0.0001	0.17	0.35	0.01	0.007	< 0.0001	0.01	0.005
iPTH (pg/ml)	BL	582 (11)	859 (79)	695 (42)	640 (29)	574 (20)	540 (18)	501 (22)	450 (52)
	Q1	575 (11)	760 (80)^a^	682 (42)	649 (29)	569 (20)	549 (18)	475 (22)	400 (52)
	Q2	586 (11)	765 (79)^a^	715 (42)	643 (29)	587 (20)	543 (18)	515 (22)	431 (52)
	*P*^d^	0.18	0.02	0.3	0.85	0.29	0.75	0.02	0.17
Calcium (mg/dl)	BL	9.10 (0.01)	9.10 (0.07)	9.07 (0.04)	9.07 (0.03)	9.09 (0.02)	9.11 (0.02)	9.15 (0.03)	9.07 (0.05)
	Q1	9.07 (0.01)^c^	9.10 (0.07)	9.05 (0.04)	9.05 (0.03)	9.05 (0.02)^a^	9.06 (0.02)^b^	9.14 (0.03)	9.11 (0.05)
	Q2	9.00 (0.01)^c^	9.09 (0.07)	8.98 (0.04)^a^	8.95 (0.03)c	9.01 (0.02)^c^	8.99 (0.02)^c^	9.05 (0.03)^c^	9.11 (0.06)
	*P*	< 0.0001	0.94	0.008	< 0.0001	< 0.0001	< 0.0001	< 0.0001	0.52
PB pills/day	BL	7.6 (0.05)	8.1 (0.30)	8.2 (0.16)	7.8 (0.11)	7.8 (0.09)	7.4 (0.08)	7.0 (0.12)	5.9 (0.19)
	Q1	4.3 (0.05)^c^	4.6 (0.29)^c^	4.7 (0.16)^c^	4.6 (0.11)^c^	4.4 (0.09)^c^	4.1 (0.08)^c^	4.0 (0.12)^c^	3.7 (0.18)^c^
	Q2	4.4 (0.05)^c^	4.9 (0.30)^c^	5.0 (0.16)^c^	4.7 (0.11)^c^	4.6 (0.09)^c^	4.1 (0.08)^c^	4.1 (0.12)^c^	4.0 (0.19)^c^
	*P*^d^	< 0.0001	< 0.0001	< 0.0001	< 0.0001	< 0.0001	< 0.0001	< 0.0001	< 0.0001
PB pills/day in in-range patients^e^	BL	6.7 (0.10)	8.6 (0.62)	7.3 (0.43)	6.6 (0.31)	6.8 (0.18)	6.6 (0.16)	6.8 (0.26)	5.0 (0.30)
	Q1	4.1 (0.08)^c^	5.0 (0.52)^b^	4.9 (0.35)^c^	4.4 (0.25)^c^	4.2 (0.15)^c^	4.1 (0.14)^c^	3.7 (0.23)^c^	3.6 (0.24)^b^
	Q2	4.2 (0.08)^c^	4.7 (0.49)^c^	5.1 (0.34)^c^	4.7 (0.25)^c^	4.3 (0.14)^c^	4.0 (0.13)^c^	4.0 (0.23) ^c^	3.6 (0.25)^b^
	*P*^d^	< 0.0001	0.0001	< 0.0001	< 0.0001	< 0.0001	< 0.0001	< 0.0001	0.0002

Values are presented as least-squared mean (standard error) for continuous variables and percentage for categorical variables

^a^*P* < 0.05

^b^*P* < .001

^c^*P* < 0.0001 (vs. BL)

^d^*P* values were calculated by mixed effects model for continuous variables and Cochran's *Q* test for categorical variables

^e^In-range patients were patients with sP ≤ 5.5 mg/dl

### PB pill burden before and after SO across age

**Fig. 3:**
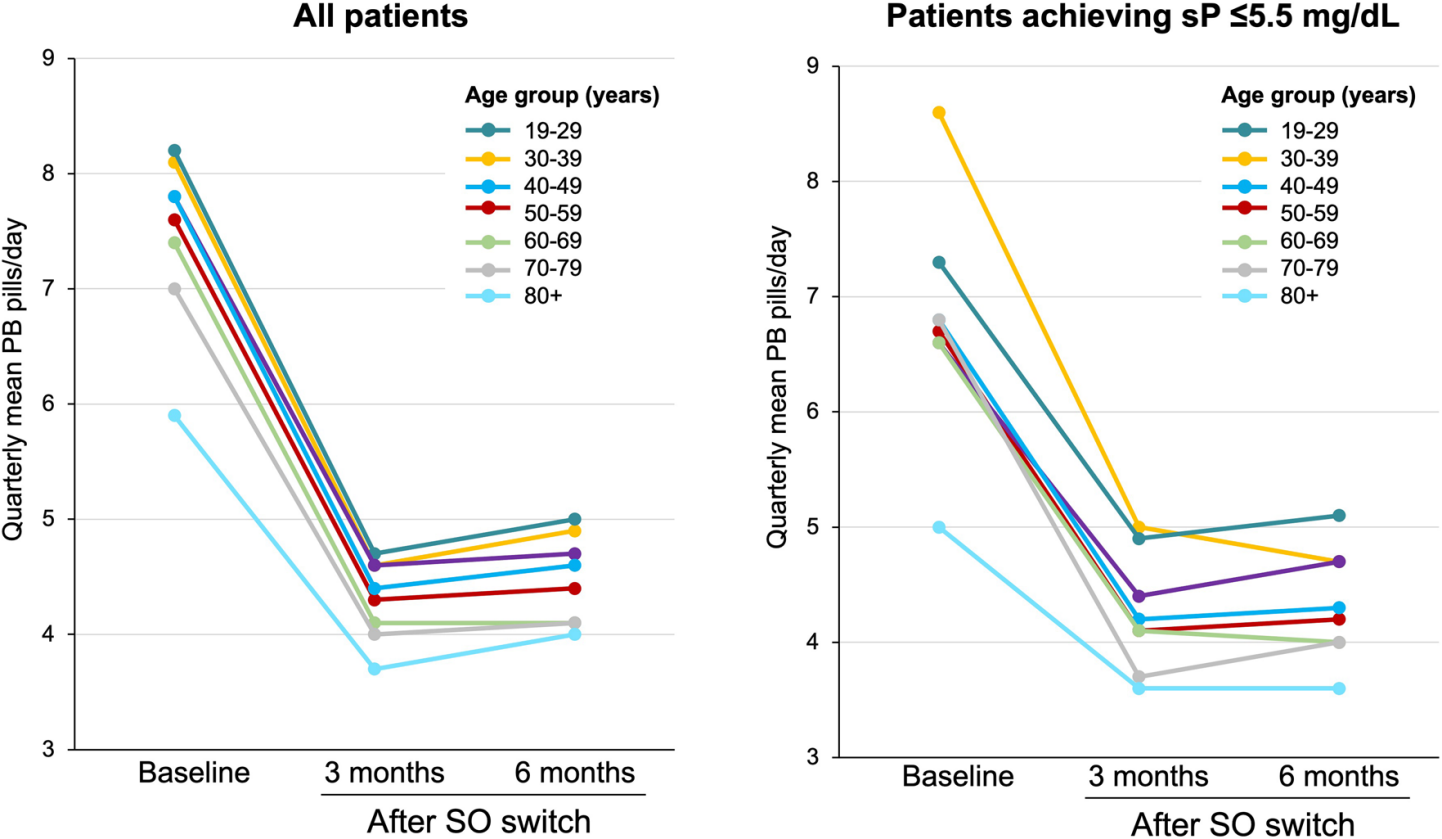
Mean number of PB pills/day at baseline and quarterly after SO switch. The prescription of SO was associated with significant reductions in the mean PB pill burden across all age groups (*P* < 0.001 vs. baseline at all time points) in both the overall population (left) and among those who achieved sP ≤ 5.5 mg/dl (right)

At baseline, younger patients had a higher pill burden compared to their older counterparts. The highest pill burden at baseline was observed among patients aged 30–39 years (mean of 8.2 PB pills/day), while the lowest was reported among those aged 80 years and older (mean 5.9 PB pills/day). After switching to SO, in the overall cohort, the mean daily PB burden decreased from a mean of 7.6 pills/day to 4.3 per day at month 3 (*P* < 0.0001), representing a 43% relative reduction in mean daily PB pill burden. Significant reductions in daily PB pill burden after switching to SO were observed across all age groups and were observed in the overall population as well as in those who achieved sP ≤ 5.5 mg/dl (Table 2, Fig. 3).

### Other MBD and nutritional parameters before and after SO across age

At baseline, serum calcium levels tended to be similar across groups, whereas serum iPTH levels were higher among patients of younger ages. Over the 6-month follow-up period, statistically significant decreases in serum calcium that remained in the normocalcemic range were observed overall and in all age subgroups except the youngest (19–29 years) and oldest (≥ 80 years) groups (Table 2). Statistically significant reductions in serum iPTH were noted only among patients aged 19–29 years.

At baseline, mean serum albumin levels were slightly higher in younger vs. older age groups (mean serum albumin 3.96 vs. 3.83 g/dl in patients 19–29 vs. ≥ 80 years old, respectively). After switching to SO, a statistically significant increase in mean serum albumin was observed during Q2 among patients younger than 60 years of age (Table 3). When adjusted for sP (i.e., phosphorus-attuned albumin), significant increases were observed across all age groups. At baseline, the lowest pre-dialysis weights were observed in patients 19–29 vs. ≥ 80 years old. After switching to SO, small but statistically significant increases in pre-dialysis weight from baseline to Q2 were observed in all age groups from 19 to 69 years, whereas pre-dialysis weight decreased in age group of ≥ 80 years old. Minimal changes were observed after SO prescription in nPCR and spKt/V.

**Table 3:** Comparison of changes in nutritional and clearance parameters among age groups

Measure	Period	All patients (*n* = 2017)	19–29 years (*n* = 65)	30–39 years (*n* = 169)	40–49 years (*n* = 362)	50–59 years (*n* = 542)	60–69 years (*n* = 553)	70–79 years (*n* = 247)	80+ years (*n* = 79)
Serum albumin (g/dl)	BL	3.87 (0.01)	3.96 (0.04)	3.92 (0.02)	3.87 (0.02)	3.89 (0.01)	3.87 (0.01)	3.78 (0.02)	3.83 (0.03)
	Q1	3.89 (0.01)^c^	4.01 (0.04)^a^	3.98 (0.02)^b^	3.89 (0.02)^a^	3.91 (0.01)^a^	3.89 (0.01)^a^	3.79 (0.02)	3.82 (0.03)
	Q2	3.89 (0.01)^c^	4.05 (0.04)^b^	3.97 (0.02)	3.90 (0.02)^b^	3.91 (0.01)	3.87 (0.01)	3.79 (0.02)	3.81 (0.03)
	*P*^d^	< 0.0001	0.001	0.0002	0.003	0.01	0.005	0.34	0.49
Phosphorus-attuned albumin (× 10^3^)	BL	0.65 (0.004)	0.60 (0.02)	0.61 (0.01)	0.61 (0.01)	0.64 (0.01)	0.67 (0.01)	0.70 (0.01)	0.72 (0.02)
	Q1	0.69 (0.004)^c^	0.63 (0.02)^a^	0.64 (0.01)^a^	0.65 (0.01)^c^	0.69 (0.01)^c^	0.71 (0.01)^c^	0.73 (0.01)^b^	0.79 (0.02)^b^
	Q2	0.71 (0.004)^c^	0.66 (0.02)^c^	0.64 (0.01)^b^	0.66 (0.01)^c^	0.71 (0.01)^c^	0.74 (0.01)^c^	0.74 (0.01)^c^	0.79 (0.02)^c^
	*P*^d^	< 0.0001	0.0004	0.0006	< 0.0001	< 0.0001	< 0.0001	< 0.0001	< 0.0001
Single pool nPCR (g/kg/d)	BL	1.02 (0.005)	1.02 (0.03)	1.02 (0.02)	1.01 (0.01)	1.03 (0.01)	1.02 (0.01)	1.00 (0.01)	1.04 (0.03)
	Q1	1.02 (0.005)	1.03 (0.03)	1.04 (0.02)	1.02 (0.01)	1.03 (0.01)	1.02 (0.01)	0.99 (0.01)	1.05 (0.03)
	Q2	1.01 (0.005)	1.05 (0.03)	1.04 (0.02)	1.01 (0.01)	1.02 (0.01)^a^	1.00 (0.01)^a^	1.00 (0.01)	1.04 (0.03)
	*P*^d^	0.03	0.36	0.32	0.22	0.04	0.006	0.85	0.73
Pre-dialysis weight (kg)	BL	91.2 (0.6)	80.7 (3.5)	93.7 (2.4)	99.2 (1.5)	93.5 (1.1)	90.6 (1.0)	82.3 (1.3)	75.4 (2.0)
	Q1	91.5 (0.6)^c^	81.1 (3.5)^c^	93.9 (2.4)^c^	99.4 (1.5)^c^	93.8 (1.1)^c^	90.9 (1.0)^c^	82.3 (1.3)	75.3 (2.0)
	Q2	91.5 (0.6)^c^	81.2 (3.5)^c^	94.1 (2.4)^c^	99.7 (1.5)^c^	93.7 (1.1)^c^	91.0 (1.0)^c^	82.3 (1.3)	75.2 (2.0)^c^
	*P*^d^	< 0.0001	< 0.0001	< 0.0001	< 0.0001	< 0.0001	< 0.0001	0.44	< 0.0001
Single pool Kt/V	BL	1.69 (0.006)	1.74 (0.04)	1.64 (0.02)	1.62 (0.02)	1.67 (0.01)	1.72 (0.01)	1.74 (0.02)	1.87 (0.03)
	Q1	1.70 (0.006)	1.76 (0.04)	1.62 (0.02)	1.64 (0.02)	1.68 (0.01)	1.72 (0.01)	1.77 (0.02)^a^	1.89 (0.03)
	Q2	1.69 (0.006)	1.77 (0.04)	1.65 (0.02)	1.64 (0.02)	1.66 (0.01)	1.71 (0.01)	1.76 (0.02)	1.85 (0.03)
	*P*^d^	0.28	0.67	0.1	0.26	0.24	0.49	0.04	0.23

Values are presented as least-squared mean (standard error) for continuous variables and percentage for categorical variables

^a^*P* < 0.05

^b^*P* < 0.001

^c^*P* < 0.0001 (vs. BL)

^d^*P* values were calculated by mixed effects model for continuous variables and Cochran's *Q* test for categorical variables

### Other clinically relevant parameters before and after SO across age

Changes in calcimimetics and/or active vitamin D analogs, iron parameters, and anemia therapies used during the follow-up period are detailed in the supplemental tables (Tables S1 and S2). Mean ferritin increased in the overall cohort (from 998 ng/ml at baseline to 1087 ng/ml, *P* < 0.0001), with the most significant increases noted among patients aged 30–69 years. Small but statistically significant reductions in the proportion of patients receiving IV iron therapy, as well as reductions in IV iron and pegylated epoetin beta dose, were observed in the overall cohort during the follow-up period.

## Discussion

In this large, contemporary cohort of US dialysis patients, we observed several age-related differences among dialysis patients. The age distribution of our population followed current aging trends observed in other dialysis studies, with 44% of patients 60 years or older and 16% of patients 70 years or older. Diabetes accounted for the largest proportion of dialysis burden in our population overall. This is consistent with recent data from the Global Burden of Disease Study, which attributed nearly a third of disability-adjusted life years (DALYs) to diabetic nephropathy [33]. While the burden of diabetes was low among younger patients, the prevalence increased substantially with age.

Similar to previous studies [5–7], older patients in our cohort were more likely to have worse nutritional parameters than their younger counterparts. Patients older than 60 years of age had lower mean sP at baseline than younger patients, a difference that was most pronounced in those aged 70 and older. Previous data have indicated that sP levels decline with age, potentially related to declining protein and caloric intake [6, 34]. Mean serum albumin and iPTH at baseline declined with age in our cohort, consistent with findings reported in previous studies [4, 34]. Patients older than 70 years had lower pre-dialysis weight and BMI compared with younger patients. Collectively, these findings reinforce the need to address inadequate dietary protein intake and hypoalbuminemia in elderly hemodialysis patients, important risk factors for protein-energy wasting and mortality in this population [6, 11, 34, 35].

The types of PBs used in our cohort varied by age, with double the proportion of patients in the youngest age group (19–29 years) prescribed calcium acetate vs. sevelamer (49.2 vs 26.2%, respectively). This difference may reflect the historic role of calcium-based PBs as the mainstay for hyperphosphatemia in pediatric and adolescent patients [36, 37], the widespread experience with these agents in younger patients [38–40], and/or recognition of the higher calcium requirements of the growing skeleton [18, 41]. Additionally, while the most recent 2017 KDIGO guidelines for CKD-MBD advise restriction of calcium-based PBs in adults, among children they advise selection of phosphate-lowering treatment according to serum calcium levels [18]. However, increasing evidence supports the safety and efficacy of non-calcium-based PBs in pediatric and adolescent patients, including sevelamer carbonate [38, 42], sevelamer hydrochloride [36], and SO [39], particularly given concerns for arterial calcification even in these younger age groups [41]. The use of lanthanum carbonate was low among the youngest group in our cohort (3.1%), a finding consistent with concern for accumulation in bone and growth impairment in pediatric patients. [43]

In addition to describing age-related patient characteristics, this study is the first to explore the real-world impact of SO prescription across various age ranges in a large cohort of hemodialysis patients. Overall, SO prescription was associated with a 62% increase in the proportion of patients reaching sP ≤ 5.5 mg/dl with a concomitant 42% reduction in daily pill burden within 6 months. Patients across all age groups experienced significant reductions in sP and pill burden after SO prescription. This benefit was most pronounced among younger patients (aged 19–29 years), in whom SO prescription was associated with a 113% relative increase in the proportion of patients achieving target sP levels. This improvement was accompanied by a reduction from 8.1 pills per day at baseline to 4.9 pills per day (*P* < 0.0001) after 6 months of follow-up.

Improvement in sP control and reduction in pill burden with SO therapy have been reported in both clinical trial [20, 27, 28, 44] and real-world settings [29–31]. In a large, active-controlled phase 3 study in patients on hemodialysis or peritoneal dialysis, an average daily dose of 3 SO tablets achieved comparable reductions in sP at 12 weeks to 8 daily sevelamer tablets [28]. A retrospective analysis of 1029 in-center hemodialysis patients demonstrated that the number of patients achieving sP ≤ 5.5 ng/ml doubled after prescription of SO, despite a 60% reduction in the daily PB pill burden [29]. Similarly, Kendrick et al. found that switching to SO led to a twofold greater probability of achieving target sP levels over a 1-year follow-up period, while halving the daily PB pill burden. [31]

Prescription of SO in our cohort was associated with statistically significant improvements in serum albumin across almost all age groups (i.e., patients aged 60 years and younger). In a previous study in 79 in-center hemodialysis patients, SO therapy was associated with significant increases in serum albumin in hypoalbuminemic patients (*P* < 0.0001), although serum albumin was unchanged in non-hypoalbuminemic patients [45]. The largest improvement in serum albumin associated with SO prescription in our study occurred in younger patients, a group that was not hypoalbuminemic based on mean serum albumin at baseline. These findings suggest that SO therapy may allow patients to increase their dietary intake of protein without compromising their serum phosphate control nor increasing their pill burden. Although there are no direct data suggesting that SO directly affects protein handling, it has been proposed that SO may allow for improved nutritional intake owing to its lower pill burden and consequent impact on appetite relative to regimens with higher pill burden [45, 46].

It is notable that at baseline, younger patients (≤ 40 years) in our cohort were prescribed an average of 8 PB pills per day but were less likely to have sP ≤ 5.5 mg/dl than older patients. This finding is consistent with previous reports of an inverse relationship between pill burden and sP levels [24–26], and underscores the potential relationship of pill burden to nonadherence with PB medications. In an analysis of pharmacy management program data involving 8616 hemodialysis patients, adherence to PB monotherapy was negatively related to higher pill burden and was significantly associated with higher mean sP levels [26]. The association between adherence and achieving target sP levels was most pronounced in patients with the highest pill burden (> 15 pills/day).

Along with reinforcing the inverse relationship between PB pill burden and serum phosphorus levels [24–26], our observations extend previous associations of nonadherence to PB medications with younger age [47–50]. Systematic reviews of studies have identified younger age as the most common patient-related factor associated with poor adherence to PB medications [47, 50]. It has been suggested that compared with older patients, younger patients may prioritize other activities over their health, may be less concerned about their mortality, may have more difficulty accepting having a chronic condition, or simply may be more likely to report nonadherence than older patients [49]. Given the pronounced improvements in mineral bone disease and statistically significant increases of nutritional parameters observed with SO prescription over a 6-month period in this real-world analysis, future research is needed to determine the longitudinal impact of SO on improving the cardiovascular health and longevity of younger dialysis patients as a particularly high-risk population.

The need to simplify and streamline PB medication regimens in hemodialysis patients is becoming increasingly important with growing evidence linking phosphate control to clinically relevant benefits in this population. Observational studies have found PB medications to improve survival in dialysis patients [51–53], but controlled trials demonstrating improved outcomes of these interventions in patients on dialysis have not been available until recently [12, 18, 54]. Analysis of data from 115 stable hemodialysis patients enrolled in the randomized, interventional EPISODE trial demonstrated that achieving strict phosphate control (i.e., to 3.5–4.5 mg/dl) with noncalcium-based phosphate binders for 12 months delayed the progression of coronary artery calcification, even among elderly patients [54]. Importantly, small differences (< 1 mg/dl) in phosphorus levels affected coronary artery calcification (CAC) scores, suggesting that even modest differences in serum phosphate may have relevant clinical benefits [13]. While short-term serum albumin improvements were not observed in the eldest age groups (i.e., ≥ 70 years old) despite substantial reductions in pill burden, further studies examining longer term effects of SO on the cardiovascular and nutritional health of this fast-growing segment of the dialysis population are warranted.

Although this analysis provides important insight into the influence of age on patient characteristics and sP management with SO, our findings should be interpreted in light of the retrospective, observational nature of the study. Other limitations include the lack of a comparator group and the potential for errors or missing relevant information in the clinical records used for data extraction. We did not collect information regarding the reasons that baseline PBs were discontinued and SO prescribed, nor did we capture data regarding safety or tolerability. Although prescription data provide meaningful information regarding pill burden, we cannot exclude the possibility that patients obtained their medication through pharmacy services other than those we reviewed. Accordingly, prescription data cannot be considered a surrogate for actual patient adherence with prescribed regimens. Many factors may influence patients’ nutritional parameters, thus observations of improvements in nutritional parameters after switch to SO should be confirmed in controlled clinical studies.

In conclusion, the results of this study complement previous findings by describing patient characteristics and nutritional parameters across varying age groups in a large, real-world population of hemodialysis patients. Serum phosphorus declined with age, and nutritional parameters were generally worse in older patients compared with younger patients. In contrast, younger patients tended to have higher sP and higher PB pill burden than their older counterparts. Additionally, switching from other PBs to SO monotherapy was associated with improved sP control and reduced pill burden in hemodialysis patients across all age groups in real-world, clinical practice. This benefit was particularly pronounced among younger patients, in whom adherence to medications such as PBs can be particularly challenging.

## Supplementary Information

Below is the link to the electronic supplementary material.

Supplementary file1 (DOCX 40 KB)
