# Supplementary material for: A real-world analysis of the influence of age on maintenance hemodialysis patients: managing serum phosphorus with sucroferric oxyhydroxide as part of routine clinical care
Source: Int Urol Nephrol. 2022 Aug 11;55(2):377–87. doi: 10.1007/s11255-022-03327-w (PMC9859895; doi:10.1007/s11255-022-03327-w)
Supplement: Supplementary file 1 — Supplementary file1 (DOCX 40 KB) [file 11255_2022_3327_MOESM1_ESM.docx]

**Table S1. Comparison of changes in MBD medications among age groups**

| **Measures** | **Period** | **All patients (n=2017)** | **19-29 y  (n=65)** | **30-39 y  (n=169)** | **40-49 y (n=362)** | **50-59 y  (n=542)** | **60-69 y  (n=553)** | **70-79 y  (n=247)** | **80+ y (n=79)** | |
| --- | --- | --- | --- | --- | --- | --- | --- | --- | --- | --- |
| Cinacalcet use (%) | BL | 27.7 | 24.6 | 25.4 | 29.8 | 27.9 | 28.6 | 26.7 | 20.3 | |
|  | Q1 | 26.2^a^ | 29.2 | 28.4 | 27.6 | 26.8 | 26^a^ | 22.7^a^ | 20.3 | |
|  | Q2 | 25.7^a^ | 30.8 | 26.6 | 26.8 | 26.4 | 25.1^a^ | 23.9 | 20.3 | |
|  | *P*^d^ | 0.005 | 0.34 | 0.45 | 0.08 | 0.45 | 0.01 | 0.07 | 1 | |
| Cinacalcet dose (mg/day) | BL | 52.6 (1.4) | 54.4 (6.6) | 55.7 (5.3) | 56.0 (3.5) | 54.3 (2.5) | 51.5 (2.6) | 44.1 (3.4) | 47.4 (9.4) | |
|  | Q1 | 54.6 (1.4)^c^ | 58.3 (6.4) | 56.7 (5.2) | 58.8 (3.6)^a^ | 56.1 (2.6)^a^ | 53.3 (2.6)^a^ | 45.7 (3.5) | 49.0 (9.4) | |
|  | Q2 | 57.1 (1.4)^c^ | 62.5 (6.4)^a^ | 59.4 (5.3)^a^ | 64.5 (3.6)^c^ | 57.4 (2.6)^b^ | 55.5 (2.6)^c^ | 47.2 (3.5)^a^ | 49.7 (9.4) | |
|  | *P*^d^ | <0.0001 | 0.13 | 0.0501 | <0.0001 | 0.0007 | <0.0001 | 0.03 | 0.2 | |
| Total vitamin D use (%) | BL | 83.1 | 89.2 | 87 | 85.4 | 81 | 83.9 | 79.8 | 79.7 | |
|  | Q1 | 83.7 | 87.7 | 86.4 | 86.2 | 82.3 | 85 | 80.6 | 73.4^a^ | |
|  | Q2 | 84.8^a^ | 87.7 | 88.8 | 86.2 | 83.8 | 86.6 | 80.6 | 74.7 | |
|  | *P*^d^ | 0.03 | 0.88 | 0.47 | 0.81 | 0.11 | 0.09 | 0.87 | 0.1 | |
| IV vitamin D use (%) | BL | 23.6 | 26.2 | 32.5 | 22.4 | 23.8 | 21 | 23.5 | 26.6 | |
|  | Q1 | 23.9 | 27.7 | 32 | 22.4 | 23.2 | 23a | 23.5 | 24.1 | |
|  | Q2 | 23.9 | 27.7 | 32.5 | 22.7 | 23.8 | 22.4 | 21.9 | 26.6 | |
|  | *P*^d^ | 0.76 | 0.72 | 0.89 | 0.96 | 0.77 | 0.06 | 0.34 | 0.61 | |
| IV doxercalciferol dose (mcg/month) | BL | 43.2 (1.4) | 48.9 (6.4) | 46.7 (4.1) | 42.9 (3.1) | 42.7 (2.5) | 41.4 (3.0) | 45.4 (4.2) | 38.6 (5.4) | |
|  | Q1 | 46.4 (1.4)^c^ | 54.6 (6.4) | 56.8 (4.1)^c^ | 44.2 (3.1) | 43.0 (2.5) | 48.4 (2.9)^c^ | 44.0 (4.2) | 34.2 (5.5) | |
|  | Q2 | 46.4 (1.4)^c^ | 49.9 (6.3) | 56.0 (4.1)^b^ | 47.0 (3.1)^a^ | 45.3 (2.5) | 47.2 (2.9)^b^ | 40.6 (4.2)^a^ | 33.5 (5.4) | |
|  | *P*^d^ | <0.0001 | 0.21 | <0.0001 | 0.03 | 0.15 | <0.0001 | 0.09 | 0.16 | |
| Oral vitamin D use (%) | BL | 61.9 | 64.6 | 56.2 | 65.7 | 60.5 | 64.7 | 57.9 | 55.7 | |
|  | Q1 | 61.7 | 63.1 | 56.8 | 66 | 60.9 | 64.4 | 57.9 | 49.4^a^ | |
|  | Q2 | 62.6 | 60 | 57.4 | 66 | 62.5 | 65.5 | 59.1 | 50.6 | |
|  | *P*^d^ | 0.37 | 0.42 | 0.87 | 0.98 | 0.29 | 0.7 | 0.7 | 0.07 | |
| Oral calcitriol dose (mcg/d) | BL | 0.89 (0.02) | 0.93 (0.10) | 1.12 (0.06) | 1.03 (0.04) | 0.88 (0.03) | 0.81 (0.03) | 0.84 (0.04) | 0.67 (0.06) | |
|  | Q1 | 0.91 (0.02) | 1.06 (0.10)^b^ | 1.05 (0.06)^a^ | 1.01 (0.04) | 0.91 (0.03)^a^ | 0.83 (0.03)^a^ | 0.86 (0.04) | 0.71 (0.06) | |
|  | Q2 | 0.95 (0.02)^c^ | 1.19 (0.10)^c^ | 1.00 (0.06)^c^ | 1.06 (0.04) | 0.93 (0.03)^b^ | 0.89 (0.03)^c^ | 0.91 (0.04)^b^ | 0.74 (0.06)^a^ | |
|  | *P*^d^ | <0.0001 | <0.0001 | 0.0004 | 0.02 | 0.0006 | <0.0001 | 0.001 | 0.08 | |
| Values are presented as least-squared mean (standard error) for continuous variables and percentage for categorical variables.  ^a^*P* <0.05; ^b^*P* <0.001; ^c^*P* <0.0001 (vs. BL) | | | | | | | | | |  |
| ^d^*P* values were calculated by mixed effects model for continuous variables and Cochran's Q test for categorical variables. | | | | | | | | | | |

**Table S2. Comparison of changes in anemia/iron indices and anemia therapies among age groups**

| **Measures** | **Period** | **All patients (n=2017)** | **19-29 y  (n=65)** | **30-39 y  (n=169)** | **40-49 y (n=362)** | **50-59 y (n=542)** | **60-69 y  (n=553)** | **70-79 y (n=247)** | **80+ y (n=79)** |
| --- | --- | --- | --- | --- | --- | --- | --- | --- | --- |
| **Anemia and iron indices** | | | | | | | | | |
| Ferritin (ng/mL) | BL | 998 (11) | 962 (50) | 911 (36) | 951 (25) | 939 (19) | 1038 (21) | 1128 (38) | 1150 (59) |
|  | Q1 | 1037 (11)^c^ | 982 (50) | 967 (36) | 954 (25) | 1010 (19)^c^ | 1078 (21)^a^ | 1152 (38) | 1165 (59) |
|  | Q2 | 1087 (11)^c^ | 991 (50) | 1012 (36)^a^ | 1012 (25)^b^ | 1054 (19)^c^ | 1134 (21)^c^ | 1194 (38)^a^ | 1219 (59) |
|  | *P*^d^ | <0.0001 | 0.79 | 0.01 | 0.0003 | <0.0001 | <0.0001 | 0.12 | 0.21 |
| Transferrin saturation (%) | BL | 32.9 (0.2) | 33.3 (1.4) | 32.3 (0.7) | 32.3 (0.5) | 32.6 (0.4) | 33.2 (0.4) | 32.8 (0.6) | 35.9 (1.3) |
|  | Q1 | 34.2 (0.2)^c^ | 35.5 (1.4) | 33.0 (0.7) | 34.9 (0.5)^c^ | 33.6 (0.4)^a^ | 34.5 (0.4)^a^ | 34.5 (0.6)^a^ | 34.4 (1.3) |
|  | Q2 | 34.7 (0.2)^c^ | 35.8 (1.4) | 34.3 (0.7)^a^ | 34.3 (0.5)^b^ | 34.0 (0.4)^b^ | 35.5 (0.4)^c^ | 34.5 (0.6)^a^ | 36.0 (1.3) |
|  | *P*^d^ | <0.0001 | 0.15 | 0.02 | <0.0001 | 0.002 | <0.0001 | 0.004 | 0.32 |
| Hemoglobin (g/dL) | BL | 10.90 (0.02) | 10.54 (0.10) | 10.85 (0.09) | 10.91 (0.05) | 10.97 (0.05) | 10.93 (0.04) | 10.85 (0.04) | 10.69 (0.06) |
|  | Q1 | 10.94 (0.02)^c^ | 10.63 (0.10) | 10.98 (0.09)^c^ | 10.91 (0.05) | 11.02 (0.05)^a^ | 10.96 (0.04)^a^ | 10.87 (0.04) | 10.75 (0.06) |
|  | Q2 | 10.89 (0.02) | 10.56 (0.10) | 10.95 (0.09)^a^ | 10.88 (0.05) | 10.98 (0.05) | 10.87 (0.04)^b^ | 10.81 (0.04)^a^ | 10.73 (0.06) |
|  | *P*^d^ | <0.0001 | 0.21 | <0.0001 | 0.23 | 0.004 | <0.0001 | 0.02 | 0.24 |
| **Anemia therapy** | | | | | | | | | |
| IV iron sucrose use (%) | BL | 77.2 | 84.6 | 77.5 | 78.2 | 79.2 | 76.5 | 75.3 | 63.3 |
|  | Q1 | 77.1 | 90.8 | 81.1 | 80.4 | 76.8 | 75.2 | 74.9 | 65.8 |
|  | Q2 | 74.8^a^ | 87.7 | 75.1 | 76 | 76 | 72.7 | 74.1 | 67.1 |
|  | *P*^d^ | 0.0499 | 0.53 | 0.26 | 0.19 | 0.29 | 0.21 | 0.93 | 0.87 |
| IV iron sucrose dose (mg/month) | BL | 371 (4.9) | 350 (22) | 377 (18) | 375 (11) | 366 (10) | 366 (9) | 378 (14) | 421 (28) |
|  | Q1 | 350 (4.9)^b^ | 302 (22) | 372 (17) | 364 (11) | 352 (10) | 346 (9) | 327 (14)^a^ | 374 (29) |
|  | Q2 | 334 (5.0)^c^ | 315 (22) | 352 (18) | 329 (11)^a^ | 349 (10) | 331 (9)^a^ | 322 (14)^a^ | 307 (29)^a^ |
|  | *P*^d^ | <0.0001 | 0.27 | 0.49 | 0.003 | 0.29 | 0.01 | 0.002 | 0.007 |
| IV ESA use (%) | BL | 85.6 | 93.8 | 84.0 | 83.4 | 85.4 | 84.1 | 88.3 | 96.2 |
|  | Q1 | 86.1 | 96.9 | 83.4 | 84.3 | 83.9 | 85.7 | 89.5 | 97.5 |
|  | Q2 | 85 | 93.8 | 82.8 | 85.1 | 83.2 | 82.8 | 89.1 | 96.2 |
|  | *P*^d^ | 0.25 | 0.56 | 0.86 | 0.56 | 0.26 | 0.08 | 0.76 | 0.78 |
| IV pegylated epoetin beta dose (mcg/month) | BL | 146 (2.5) | 172 (14) | 160 (10) | 159 (6) | 144 (5) | 141 (5) | 132 (6) | 122 (9) |
|  | Q1 | 142 (2.5) | 156 (14) | 174 (10) | 157 (6) | 137 (5) | 134 (5) | 131 (6) | 120 (9) |
|  | Q2 | 136 (2.5)^c^ | 159 (14) | 170 (10) | 148 (6)^a^ | 137 (5) | 127 (5)^b^ | 119 (6)^a^ | 113 (9) |
|  | *P*^d^ | <0.0001 | 0.32 | 0.19 | 0.06 | 0.12 | 0.0006 | 0.01 | 0.48 |

Values are presented as least-squared mean (standard error) for continuous variables and percentage for categorical variables.

^a^*P* <0.05; ^b^*P* <0.001; ^c^*P* <0.0001 (vs. BL)

^d^*P* values were calculated by mixed effects model for continuous variables and Cochran's Q test for categorical variables.
